# A new lumbar posterior fixation system, the memory metal spinal system: an in-vitro mechanical evaluation

**DOI:** 10.1186/1471-2474-14-269

**Published:** 2013-09-18

**Authors:** Dennis Kok, Paul John Firkins, Frits H Wapstra, Albert G Veldhuizen

**Affiliations:** 1 Universitair Medisch Centrum Groningen, Hanzeplein 1, 9700, RB Groningen, The Netherlands; 2 DePuy Spine International, 325 Paramount Dr, Raynham, MA 02767, USA

**Keywords:** Memory metal spinal system, NiTi, DePuy spines titanium moss Miami, In-vitro mechanical evaluation, ASTM standard F1717-96

## Abstract

**Background:**

Spinal systems that are currently available for correction of spinal deformities or degeneration such as lumbar spondylolisthesis or degenerative disc disease use components manufactured from stainless steel or titanium and typically comprise two spinal rods with associated connection devices (for example: DePuy Spines Titanium Moss Miami Spinal System). The Memory Metal Spinal System of this study consists of a single square spinal rod made of a nickel titanium alloy (Nitinol) used in conjunction with connecting transverse bridges and pedicle screws made of Ti-alloy. Nitinol is best known for its shape memory effect, but is also characterized by its higher flexibility when compared to either stainless steel or titanium. A higher fusion rate with less degeneration of adjacent segments may result because of the elastic properties of the memory metal. In addition, the use of a single, unilateral rod may be of great value for a TLIF procedure. Our objective is to evaluate the mechanical properties of the new Memory Metal Spinal System compared to the Titanium Moss Miami Spinal System.

**Methods:**

An in-vitro mechanical evaluation of the lumbar Memory Metal Spinal System was conducted. The test protocol followed ASTM Standard F1717-96, “Standard Test Methods for Static and Fatigue for Spinal Implant Constructs in a Corpectomy Model.”

1. Static axial testing in a load to failure mode in compression bending,

2. Static testing in a load to failure mode in torsion,

3. Cyclical testing to estimate the maximum run out load value at 5.0 x 10^6 cycles.

**Results:**

In the biomechanical testing for static axial compression bending there was no statistical difference between the 2% yield strength and the stiffness of the two types of spinal constructs.

In axial compression bending fatigue testing, the Memory Metal Spinal System construct showed a 50% increase in fatigue life compared to the Titanium Moss Miami Spinal System.

In static torsional testing the Memory Metal Spinal System constructs showed an average 220% increase in torsional yield strength, and an average 30% increase in torsional stiffness.

**Conclusions:**

The in-vitro mechanical evaluation of the lumbar Memory Metal Spinal System showed good results when compared to a currently available spinal implant system. Throughout testing, the Memory Metal Spinal System showed no failures in static and dynamic fatigue.

## Background

Chronic low back pain can be the result of spondylolisthetic or degenerative lumbar segmental instability [1,2]. Surgical treatment of this condition by fusion of the involved segments was introduced in the mid-1920s [1,3]. Posterior lumbar interbody fusion (PLIF) has become a clinically established and increasingly popular procedure since its introduction by Cloward [4-7]. A successful PLIF can restore disc height, decompress the dural sac and nerve roots, immobilize the unstable intervertebral disc, and restore load bearing to anterior structures [8]. Rigid instrumentation was added to improve initial stability and to improve fusion rates of interbody fusion. On the other hand however, instrumented spinal fusion plays a major part in the development of adjacent segments degeneration because of increased stiffness of the fused motion segment [9-12].

We developed a system that can be used in the treatment of diseases in the lumbar region (short construct) as well as the 3-dimensial corrections of scoliotic deformities [13]. We started with the development of the short lumbar construct, to evaluate its use for lumbar degenerative conditions. There may be specific advantages of this unique system for lumbar use, and it will generate proof of concept for lumbar as well as long constructs, as used for the treatment of deformities. The Memory Metal Spinal System is a posterior system, consisting of a single spinal rod used in conjunction with pedicle screws and connecting transverse bridges. The use of a unilateral single rod with pedicle screw fixation was proposed to decrease the stiffness of the implant and would be as effective as the conventional system with two rods and bilateral pedicle fixation [14,15]. In addition, a unilateral may facilitate a TLIF procedure, because the rod will not obstruct a TLIF cage at the contra-lateral side.

Spinal systems that are currently available use components manufactured from stainless steel or titanium. Before implantation into humans the new Memory Metal Spinal System must be proven to be at least substantially equivalent in performance and safety to current deformity systems. The spinal rod component used in this system is manufactured from Nitinol (NiTi), a nickel-titanium alloy. The characteristics of this alloy were first described by Buehler and Wang [16]. NiTi is a Memory Metal and is mainly characterized by its shape memory effect. At present, the characteristics of this NiTi alloy are used clinically in wires for orthodontic tooth alignment, osteosynthesis staples, vena cava filters and other vascular applications [17-22]. Wever et al. looked at the biocompatibility and functionality of this new Memory Metal Spinal System [23]. In the experimental animal study on six pigs, nickel levels measured post-operatively were similar to the results recorded preoperatively. Corrosion and fretting processes were not observed; no adverse tissue reactions were evident.

Use of a single rod manufactured from memory metal, which offers increased elasticity compared to stainless steel or titanium and therefore ease of use, should lead to a reduction in the length of time for operation, which in turn should lead to other desirable outcomes as reduced blood loss. There should also be less degeneration of adjacent segments (adjacent level disease) and better fusion is expected because of less rigidity in the memory metal spinal system. With current systems there may be loss of achieved reposition due to the viscous properties of the spine. By using a memory metal in this new system the expectation was that there is a better maintenance of the reposition due to the metal’s inherent shape memory properties (continues reposition force).

The purpose of this study is to fully biomechanically test the Memory Metal Spinal System according to ASTM F1717-96, “Standard Test Methods for Static and Fatigue for Spinal Implant Constructs in a Corpectomy Model” [24].

We used the DePuy Spine’s Titanium Moss Miami Spinal System as a comparison control. This system has proven clinical efficacy and safety, and is used to treat similar spinal disorders, as the Memory Metal Spinal System will be used for.

## Methods

### Memory metal spinal components

#### Rods

The Spinal Rods used are manufactured from medical grade Nickel Titanium Alloy according to ASTM F2063-00 standard.

This standard references the acceptable biocompatibility of the material. The austenite start temperature (A_s_) was set between 0 & 10°C. This meant that at room and body temperature the rod was fully austenitic (A_f_) and the rod was super elastic. Being able to quote the ASTM standard allows international regulatory acceptance of the material to obtain the CE mark for the implants. The cross sectional shape of the rod is square which allows good torsional stability, and correction possibilities. A square cross-sectional rod profile may additionally allow deformity correction in the transverse plane when engaged in squared head pedicle screws or connection bridge [25].

**Figure 1:**
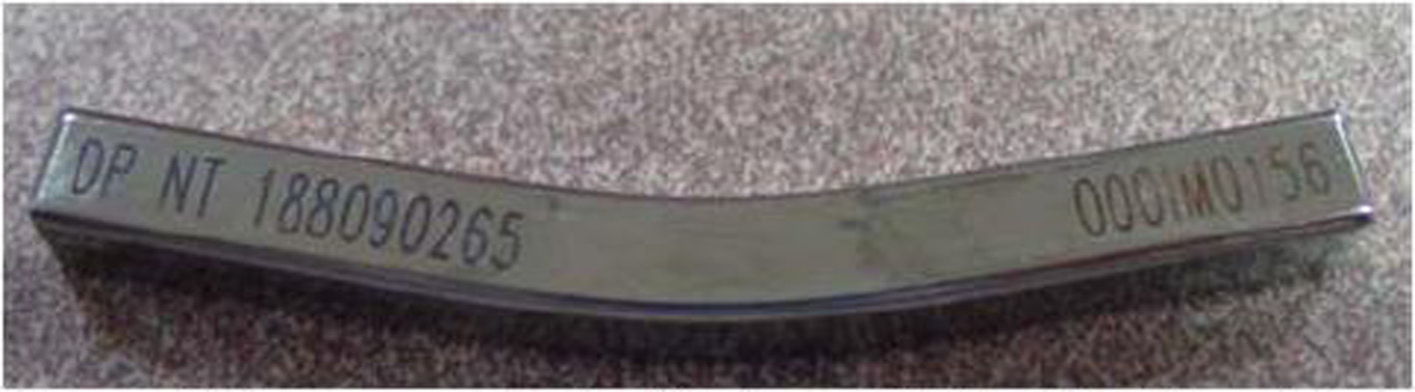
Square, anatomically shaped NiTi alloy spinal rod.

The rod has a curved shape to fit anatomically in the lumbar spine, as shown in Figure 1.

The rods have a 6.35 mm square cross-section; current systems often have a round rod of this cross section. The square design of the rod adds torsional stability within the connecting bridge. Rods are available straight or pre-bent (pre-lordosed). A surgeon using conventional systems will generally apply a bend to the rod to achieve lordosis in line with the anatomy of the spine in the lumbar region. Therefore, providing a surgeon with a pre-bent rod should make the surgical technique quicker and simpler. The length of the rods can be cut to the specific length required by the surgeon by using a specially designed cutter. During manufacturing, shape setting was done by heat treatment, and surface treatment carried out to give appropriate corrosion resistance.

#### Pedicle screws

Anchoring the spinal rod to the spine is achieved by pedicle screws. Pedicle screws have been used extensively in the lumbar region due to their strong fixation capabilities. The pedicle screws used in this study will be from DePuy Spine’s CE marked Spinal System called’Monarch’ ™. They are manufactured from a medical grade titanium alloy (Titanium Per MS-401 Grade 01).

#### Connector bridge

**Figure 2:**
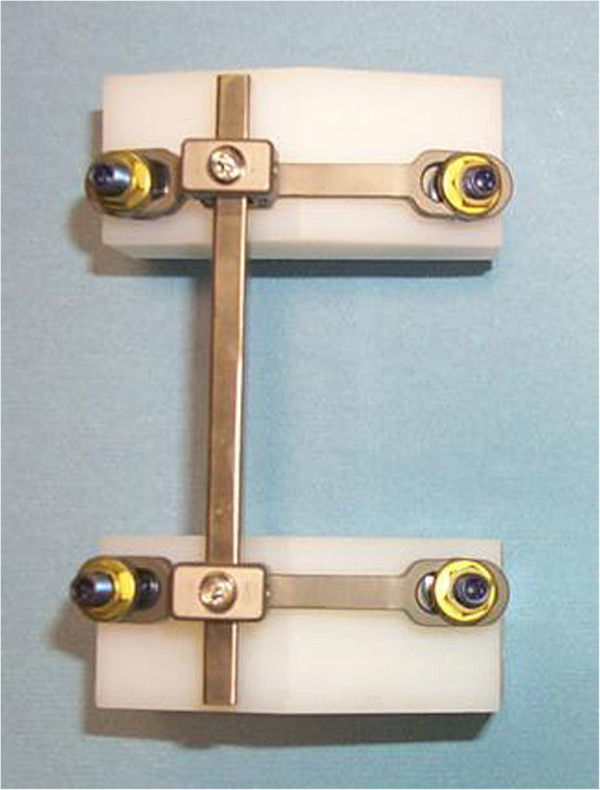
Assembled memory metal spinal system construct: posterior view.

Attached between 2 pedicle screws on one vertebra is a transverse connecting bridge, available in various sizes. Offset on the bridge is a channel, which the square spinal rod is attached to. In vivo this allows the rod to be set lateral of the spinous process. The rod is fixed in the channel by a cap and setscrew combination, as shown in Figure 2.

The transverse connecting bridge is a device which is fixed between two polyaxial pedicle screws to produce a stable, rigid construct used to hold the spinal rod in place. This rigid construct aids the transfer of correctional forces from the rod to the vertebrae. The flexibility of the system comes from the rod and not the connector or screws. The rod is connected to the bridge and locked in place by a set screw and sliding cap. The rod can be approximated to the bridge using a mini approximator instrument. Connecting bridges are available in a range of standard sizes to accommodate the range of different anatomical sizes of the human vertebrae. All connecting bridges are manufactured from medical grade Titanium Alloy which is considered safe to use with Nitinol. The simplest lumbar construct would consist of four pedicle screws, two connector bridges, and one rod, and would be implanted over two adjacent vertebral levels.

### The titanium moss Miami components

The titanium MOSS Miami System includes rods and polyaxial screws with inner screws and outer locking nuts. Bilateral constructs were assembled using the smallest size pedicle screws (5.5 mm). Only 5.5 mm rods are available in this system. These components were mounted onto two ultra high molecular weight polyethylene (UHMWPE) blocks to create a bilateral construct. The gap between the blocks ensured that no load sharing took place, i.e. the assembled bilateral MOSS Miami constructs alone resisted the applied load. Each polyaxial screw was tightened down so that the screw head was tight against the test block and then turned back 90 degrees. The construct blocks were aligned so that the ends were parallel to one another. Two 5.5 mm diameter rods were positioned into the slots of the polyaxial screws and the inner screws and outer nuts were hand tightened onto the polyaxial screws. The gauge length (the distance between the center of the upper screws to the center of the lower screws) and the moment arm from the loading point to the center of the longitudinal member were kept constant at 76.0 ±1.0 mm and 48.5 ±1.0 mm, respectively, for all constructs. A cross connector was placed halfway between the upper and lower UHMWPE blocks. The inner screws were tightened to 6 Nm, the outer nuts were tightened to 10 Nm and cross connector set screws were tightened to 6 Nm. The tightened sequence for the inner and screws/outer nuts was: 1. inner screw, 2. outer nut, 3. inner screw.

### Testing protocol

Testing was undertaken to determine the mechanical properties of the Memory Metal Spinal System compared to DePuy Spine’s Titanium Moss Miami Spinal System. If the lumbar Memory Metal Spinal System performs at least as well as the Moss Miami system in the biomechanical tests, it is assumed that the system will perform appropriately in the clinical setting from a biomechanical perspective.

The objectives were to evaluate the Memory Metal Spinal System when compared to the DePuy Spine’s Titanium Moss Miami Spinal System in the following test methods:

1. Static axial testing in a load to failure mode in compression bending,

2. Static testing in a load to failure mode in torsion,

3. Cyclical testing to estimate the maximum run out load value at 5.0 × 106 cycles.

The test protocol followed ASTM Standard F1717-96, “Standard Test Methods for Static and Fatigue for Spinal Implant Constructs in a Corpectomy Model.”

Spinal implants are generally composed of several components which, when connected together, form a spinal implant assembly. Spinal implant assemblies are designed to provide some stability to the spine while arthrodesis takes place. These test methods outline standard materials and methods for the evaluation of different spinal implant assemblies so that comparison between different designs may be facilitated. These test methods are used to quantify the static and dynamic mechanical characteristics of different designs of spinal implant assemblies. The mechanical tests are conducted *in vitro* using simplified load schemes and do not attempt to mimic the complex loads of the spine. These test methods set out guidelines for load types and methods of applying loads. Methods for three static load types and one fatigue test are defined for the comparative evaluation of spinal implant assemblies. And these test methods establish guidelines for measuring displacements, determining the yield load, and evaluating the stiffness and strength of the spinal implant assembly.

A Corpectomy model simulates a worst-case scenario for a spinal construct, i.e. the model simulates the implants being implanted in two vertebral bodies with a vertebral body missing in-between therefore loading the implants fully. Normally implants share load with the actual spinal column.

All static torsion tests were conducted using an INSTRON 8874 Bi-Axial Table Top Servohydraulic Dynamic Testing System (INSTRON, Canton, MA) with a 25 kN and 100 Nm load cell. All axial compression bending tests were conducted using an INSTRON 8872 Axial Table Top Servohydraulic Dynamic Testing System (INSTRON, Canton, MA) with a 10 kN load cell. All tests were conducted using an environmental chamber, holding Phosphate Buffered Saline at 37°C ± 3°C, for the duration of the tests. Stainless steel fixtures were used to minimize corrosion products from being generated in the test environment.

#### Static axial compression bending test

Five (5) Memory Metal Spinal System constructs and titanium 5.5 mm diameter Moss Miami constructs were tested on an INSTRON 8872 Axial Table Top Servohydraulic Dynamic Testing System in static axial compression bending to measure the compression bending yield load (N), compression bending stiffness.

(N/mm), and compression bending peak load (N). The failure mode of each construct was also recorded. The axial static compression bending tests were conducted in displacement control at a rate of 0.1 mm/sec, collecting load and displacement data. The ramp waveform was conducted until the construct experienced a permanent deformation, the test blocks touched (25 mm relative actuator displacement), or gross failure occurred. The test specimens were assembled per manufacturer instructions, and mounted in UHMWPE blocks. Statistical analysis included calculation of the mean and standard deviation of each measured value.

#### Static torsion test

Five (5) Memory Metal Spinal System constructs and titanium 5.5 mm diameter Moss Miami constructs were statically tested on an INSTRON 8874 Bi-Axial Table Top Servohydraulic Dynamic Testing System in torsion, measuring the yield torque (N-m), torsional stiffness (N-m/degree) and the peak torque (N-m). The failure mode of each construct was recorded. The test specimens were assembled per manufacturer instructions, and mounted in UHMWPE blocks (per ASTM F1717). The torsion static tests were conducted with a static axial compression preload (20 N), in angular displacement control at a rate of 1°/sec, collecting torque and angular displacement data. The ramp waveform was conducted until the construct experienced a permanent deformation or reached 80° of angular displacement. Statistical analysis included calculation of the mean and standard deviation of each measured value.

#### Dynamic axial compression bending test

Six (6) Memory Metal Spinal System constructs and titanium 5.5 mm diameter Moss Miami constructs were tested in axial compression bending fatigue using a servo hydraulic testing machine. The testing configuration matched that of the static axial compression bending tests. A cyclic load with a constant frequency of 3 Hz was applied to each construct. The loads were maintained with a constant sinusoidal load amplitude control and a constant load ratio (R = min/max) equal to 10. Load values were chosen to develop a fatigue curve with two (2) specimens reaching 5,000,000 cycles without evidence of failure. Testing was terminated when the construct experienced permanent deformation (actuator axial displacement greater than ±2 mm) or reached 5,000,000 cycles. Dynamic stiffness (Force/Displacement) was calculated during the first 2000 cycles by capturing the peak and valley values from both the force and displacement sine waves. The failure mode of each construct and the corresponding cycle count were recorded. A fatigue curve with 95% confidence limits was also generated using TableCurve 2D (Jandel Scientific, Chicago, IL).

## Results

### Static axial compression bending test

**Figure 3:**
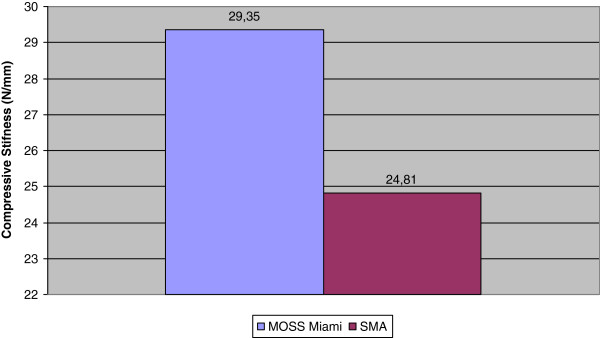
Static axial compression bending testing.

The graphics of the compression bending stiffness (N/mm) for the static axial compression bending testing of the Memory Metal Spinal System constructs and the titanium 5.5 mm diameter Moss Miami constructs are shown in Figure 3. Table 1 contains the data, mean values and standard deviations for the compression bending yield load (N), compression bending stiffness (N/mm) and compression bending peak load (N) and failure mode. The peak load is defined as the highest values attained during the testing. The specimens did not experience a fracture or gross failure. The test was stopped when the test blocks touched. However, the Memory Metal test specimens did experience rotation of the superior block about the transverse bridge. Specimens AC1, AC4, and AC5 also experienced rotation of the rod about the inferior transverse bridge, as seen by movement of the inferior washers. A typical failure for the MOSS Miami test specimen was slippage in the polyaxial connections of the screws, followed by rod deformation after the polyaxial head could not slip further.

**Table 1:** Static axial compression bending testing

**MOSS Miami**			
**Specimen**	**Compressive stifness (N/mm)**	**2% yield load (N)**	**Peak load (N)**
1	28.80	284.6	647.0
2	29.92	292.6	652.4
3	30.04	249.7	617.5
4	29.42	288.6	625.5
5	29.55	249.7	625.5
Mean:	29.35	273.0	633.6
SD:	0.50	21.50	15.20
**Memory Metal Spinal System**			
**Specimen**	**Compressive stifness (N/mm)**	**2% yield load (N)**	**Peak load (N)**
1	27.62	227.16	335.72
2	24.82	258.27	365.93
3	26.12	264.87	396.69
4	21.19	365.97	434.75
5	24.32	306.71	409.48
Mean:	24.81	284.60	388.51
SD:	2.395	53.600	38.541

### Static torsion test

**Figure 4:**
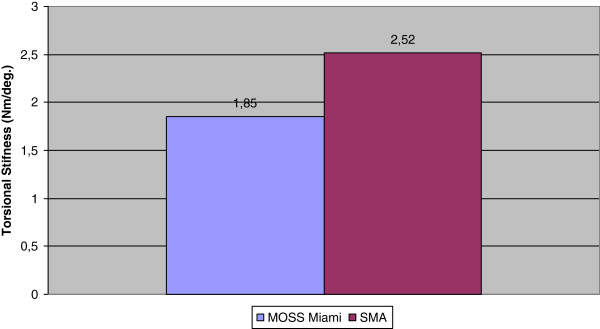
Static torsion testing.

The graphics of the compression bending stiffness (N/mm) for the static torsion testing of the Memory Metal Spinal System constructs and titanium 5.5 mm diameter Moss Miami constructs are shown in Figure 4. Table 2 contains the data, mean values and standard deviations for the yield torque (N-m), torsional stiffness (N-m/degree) and the peak torque (N-m) of each of the tested constructs. The peak torque is defined as the highest values attained during the testing. The specimens did not experience a fracture and the test was stopped when the test blocks rotated. However, the Memory Metal test specimens did experience rotation of the superior block about the transverse bridge, and the inferior transverse bridge experienced bending about the center of the test block. Typical failure for the MOSS Miami test specimens was cross-connector rod deformation at the j-hook, slippage of the open j-hooks on the rod and rotation of the polyaxial screw head.

**Table 2:** Static torsion testing

**MOSS Miami**			
**Specimen**	**Torsional stiffness (Nm/deg.)**	**2% yield torque (Nm)**	**Peak torque (Nm)**
1	1.85	20.77	25.57
2	1.77	18.62	24.83
3	1.95	20.71	25.92
4	1.89	19.75	25.55
5	1.94	19.11	25.56
Mean:	1.85	19.78	25.49
SD:	0.074	0.955	0.397
**Memory Metal Spinal System**			
**Specimen**	**Torsional stiffness (Nm/deg.)**	**2% yield torque (Nm)**	**Peak torque (Nm)**
1	2.38	61.28	74.16
2	2.59	61.66	76.46
3	2.53	65.53	76.17
4	2.63	65.14	77.81
5	2.48	66.07	77.74
Mean:	2.52	63.94	76.47
SD:	0.098	2.280	1.487

### Dynamic axial compression bending test

**Figure 5:**
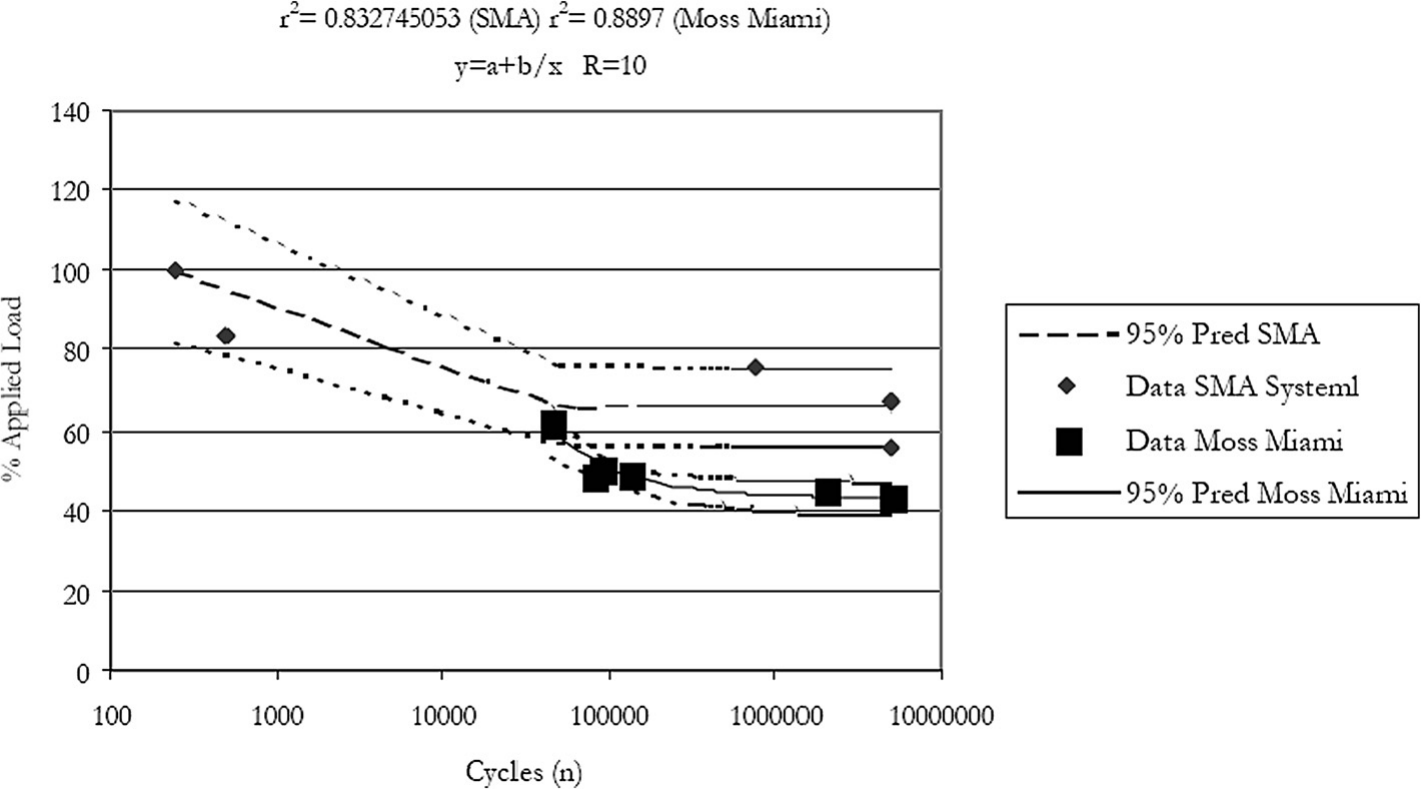
Axial compression bending fatigue curves for the memory metal spinal system (sma) & the moss Miami system.

Six (6) Memory Metal Deformity Implant System constructs and titanium 5.5 mm diameter Moss Miami constructs were tested in axial compression bending fatigue. Table 3 outlines the axial compression bending fatigue results for each specimen, including the applied load, the cycles to failure, and failure mode. The r2 value for the curve was calculated to be 0.832 (TableCurve 2D, Jandel Scientific), and is shown with 95% confidence limits in Figure 5.

**Table 3:** Dynamic axial compression bending testing

**Memory Metal Spinal System**			
**Specimen**	**Load (N)**	**Cycles to failure (n)**	**Failure mode**
1	198	5000000	No observed failure
2	237	5000000	No observed failure
3	237	5000000	No observed failure
4	269	771901	Superior screws rotate inferiorly about superior transverse bridge
5	296	500	Superior screws rotate inferiorly about superior transverse bridge
6	355	250	Superior screws rotate inferiorly about superior transverse bridge
**MOSS Miami**			
**Specimen**	**Load (N)**	**Cycles to failure (n)**	**Failure mode**
1	160	5000000	No observed failure
2	170	5000000	No observed failure
3	172	80000	Screw head/shank interface
4	175	134537	Screw head/shank interface
5	180	90811	Screw head/shank interface
6	220	44251	Screw head/shank interface

## Discussion

In the biomechanical testing for static axial compression bending there was no statistical difference between the 2% yield strength and the stiffness of the two types of spinal constructs. Even though the Memory Metal Spinal System construct consists of only one rod compared to two rods for Moss Miami. For the Memory Metal Spinal System constructs, failure occurred at the bridge/screw connection. A typical failure for the Moss Miami constructs was slippage in the polyaxial connections of the screws. The square rod and the rigid bridge/screw connections help with its overall strength. In axial compression bending fatigue testing, the Memory Metal Spinal System construct showed a 50% increase in fatigue life compared to Moss Miami. Fatigue failure for the Memory Metal Spinal System constructs failed again at the bridge/screw connection, Moss Miami construct failure was due to fracture of the screw shank. It is suspected that the super elastic property of the Memory Metal Spinal System rod relieves stress from the rest of the rigid construct.

In static torsional testing the Memory Metal Spinal System constructs showed an average 220% increase in torsional yield strength, and an average 30% increase in torsional stiffness. The former was due to the super elastic properties of the Memory Metal Spinal System rod, the latter was due to the square cross section of the Memory Metal Spinal System rod. It is important to note that in all tests no failure of the Memory Metal Spinal System rods occurred.

After these encouraging results the further development with a lumbar construct is underway and has shown clinical efficacy. Twenty-seven patients have been implanted with the simple lumbar construct for the treatment of lower back disorders. To date all patients are doing well.

The next stages in development will include, implantation of the full scoliosis construct to prove the system can stabilize the spine using the same material specification as the simple spinal construct, and finally a full scoliosis system based on the Memory Metal Spinal System generating 3D correctional forces using temperature controlled shape memory specification material. If the development is successful, this unique technology may allow the future development of a non-fusion system, which is recognized as the ultimate goal in treating patients with scoliosis.

## Conclusions

Biomechanical testing showed good results when compared to currently available spinal implant systems. Throughout testing, the Memory Metal spinal rod showed no failures in static and dynamic fatigue.

### Key points

In-vitro mechanically evaluation (ASTM Standard F1717-96) of the new lumbar Memory Metal Spinal System.

The Memory Metal spinal rod showed no failures in static and dynamic fatigue.

## Competing interests

The authors declare that they have no competing interests.

## Authors’ contributions

DK performed the testing, analyzed the data and drafted the manuscript. PF designed the testing protocol. FW participated in the design of the study. AV conceived of the study, and participated in its design and coordination. All authors read and approved the final manuscript.

## Pre-publication history

The pre-publication history for this paper can be accessed here:

http://www.biomedcentral.com/1471-2474/14/269/prepub
